# Supplementary material for: DMRforPairs: identifying Differentially Methylated Regions between unique samples using array based methylation profiles
Source: BMC Bioinformatics. 2014 May 15;15:141. doi: 10.1186/1471-2105-15-141 (PMC4046028; doi:10.1186/1471-2105-15-141)
Supplement: Additional file 2 — DMRforPairs output for the comparison of A431-MCF7 and NA17018-NA17105. Please start from the HTML files in each folder. Available via the BMC Bioinformatics website. [file 1471-2105-15-141-S2.zip › 1394847754114233_MOESM2_ESM/A431_MCF7/figures/20404.pdf]

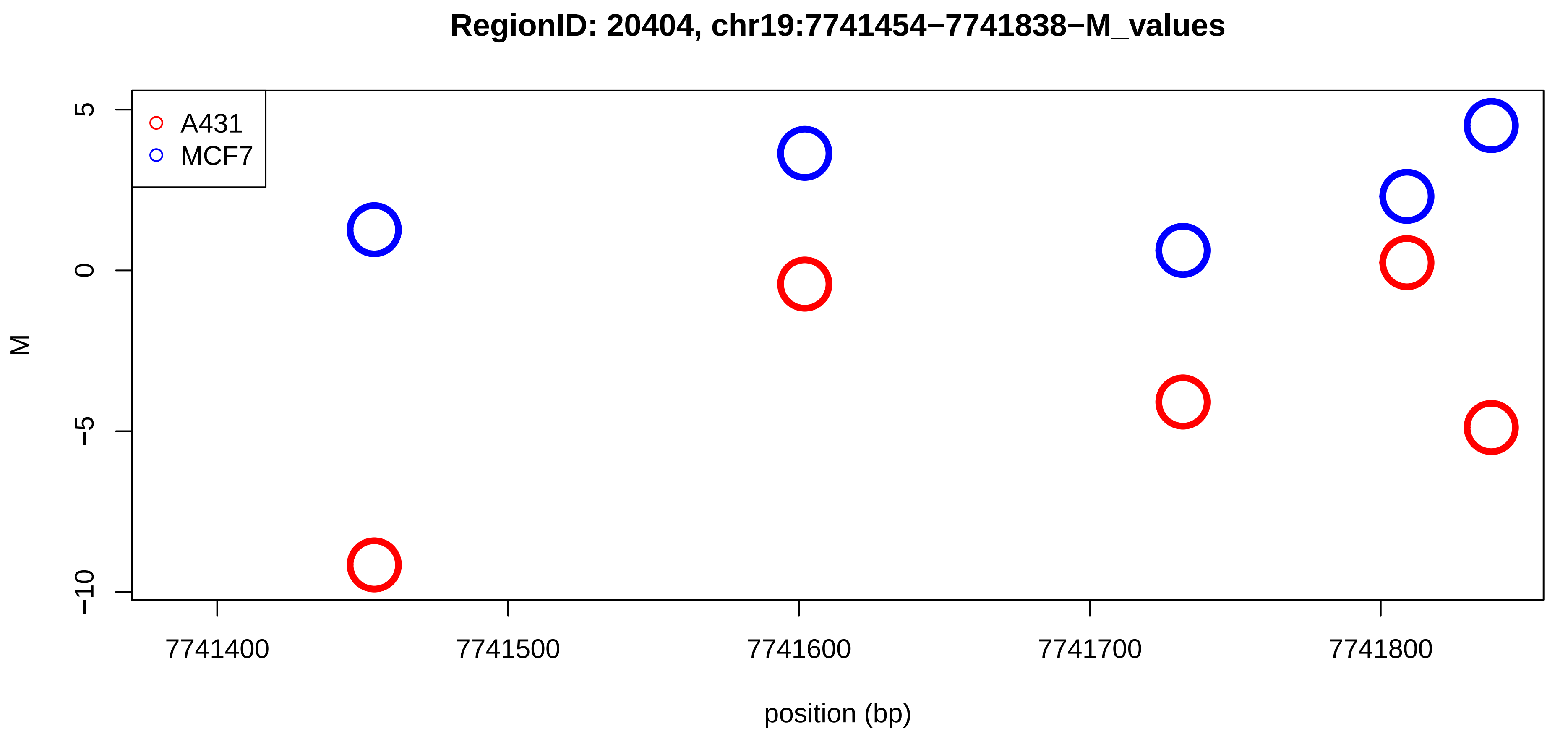
RegionID: 20404, chr19:7741454−7741838−M_values
A431
MCF7
M
5
0
−5
−10
7741400
7741500
7741600
7741700
7741800
position (bp)

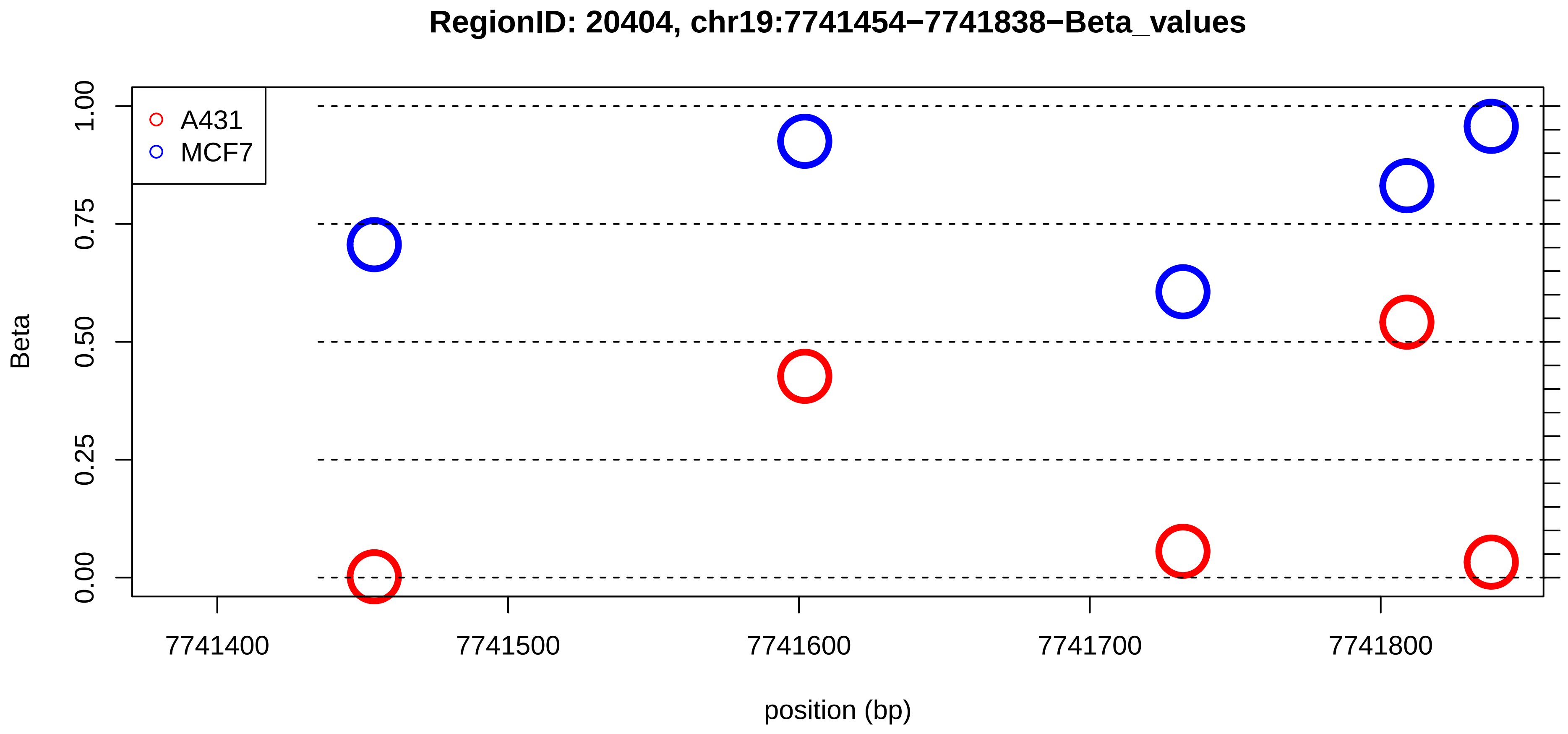
RegionID: 20404, chr19:7741454−7741838−Beta_values
A431
MCF7
Beta
1.00
0.75
0.50
0.25
0.00
7741400
7741500
7741600
7741700
7741800
position (bp)

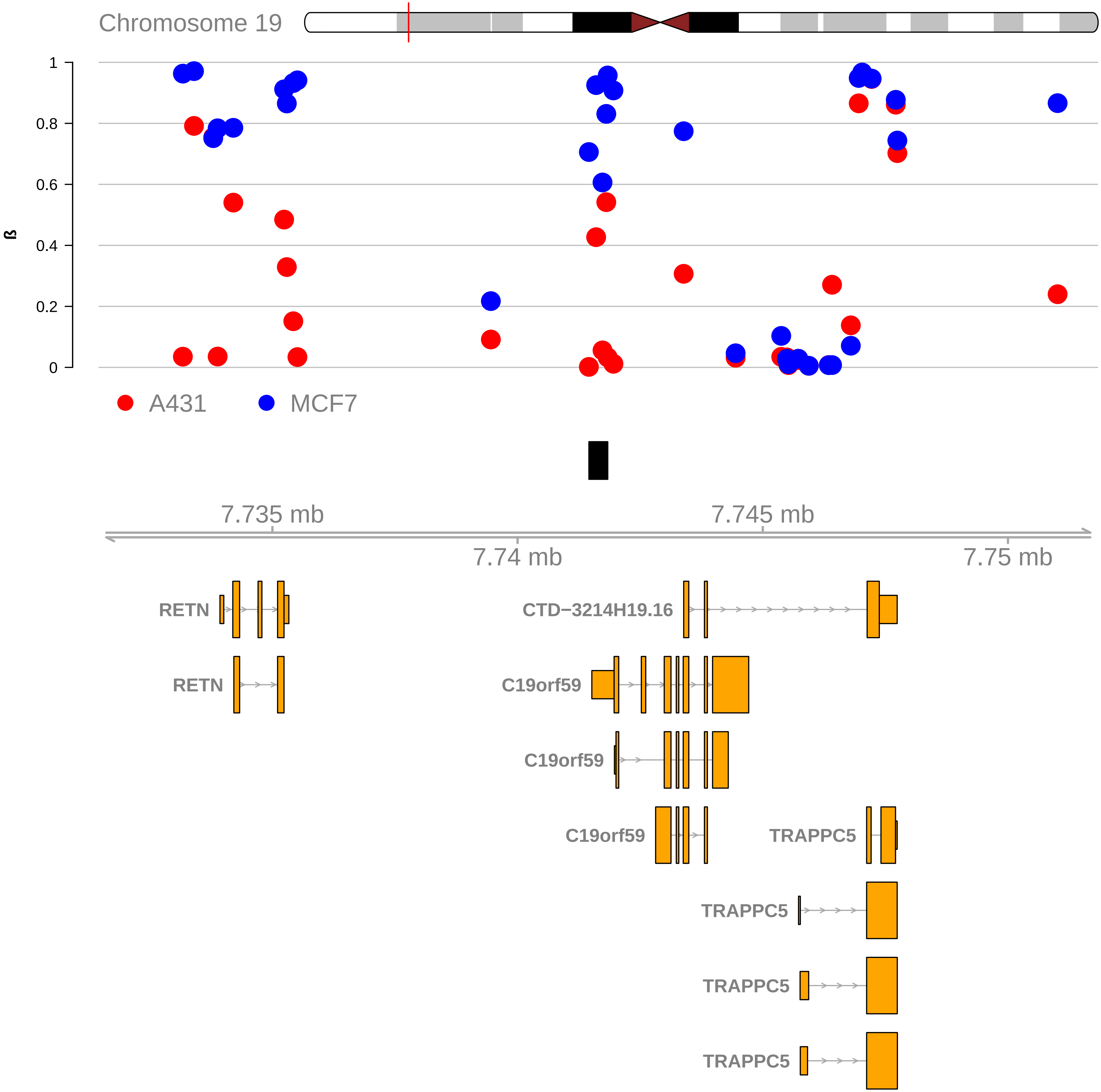
Chromosome 19
β
1
0.8
0.6
0.4
0.2
0
A431
MCF7
7.735 mb
7.745 mb
7.74 mb
7.75 mb
RETN
RETN
CTD−3214H19.16
C19orf59
C19orf59
C19orf59
TRAPPC5
TRAPPC5
TRAPPC5
TRAPPC5
